# Evaluating renewable natural resources flow and net primary productivity with a GIS-Emergy approach: A case study of Hokkaido, Japan

**DOI:** 10.1038/srep37552

**Published:** 2016-11-18

**Authors:** Chengdong Wang, Shenyan Zhang, Wanglin Yan, Renqing Wang, Jian Liu, Yutao Wang

**Affiliations:** 1 Institute of Ecology and Biodiversity, School of Biological Sciences, Jinan 250100, Shandong University, P.R. China; 2 Graduate School of Media and Governance, Keio University, Fujisawa 252-0882, Japan; 3 Institute of Environmental Research, Shandong University, Jinan, China; 4 Department of Industrial System and Engineering, University of Tennessee, Knoxville 37996, USA

## Abstract

Renewable natural resources, such as solar radiation, rainfall, wind, and geothermal heat, together with ecosystem services, provide the elementary supports for the sustainable development of human society. To improve regional sustainability, we studied the spatial distributions and quantities of renewable natural resources and net primary productivity (NPP) in Hokkaido, which is the second largest island of Japan. With the help of Geographic Information System (GIS) software, distribution maps for each type of renewable natural resource were generated by kriging interpolation based on statistical records. A composite map of the flow of all types of renewable natural resources was also generated by map layer overlapping. Additionally, we utilized emergy analysis to convert each renewable flow with different attributes into a unified unit (i.e., solar equivalent joules [sej]). As a result, the spatial distributions of the flow of renewable natural resources of the Hokkaido region are presented in the form of thematic emergy maps. Thus, the areas with higher renewable emergy can be easily visualized and identified. The dominant renewable flow in certain areas can also be directly distinguished. The results can provide useful information for regional sustainable development, environmental conservation and ecological management.

Fossil fuels are a dominant energy source supporting the development of human social economic activities. However, enormous pressure on this source has been generated by rapid increases in energy consumption^1^,^2^. Because the importance of sustainable development is now widely recognized, renewable resources as basic sources of natural capital play an important role in supporting ecosystem function and sustaining social economic development^3^,^4^.

Many studies have addressed renewable resources and natural capital, and regional sustainable development is attracting the attention of increasing numbers of scholars and governments. For example, the renewable resources and ecosystem services of the U.S. national forest system have been evaluated through environmental accounting^5^, which revealed that abundant rewards were obtained from forest ecosystems, even after subtracting the government’s budget for forest conservation. Jones *et al*. discussed the ecosystem services derived from both natural and human-derived capital and suggested that the importance of all ecosystem services should be considered in environmental policy formulation and implementation^6^. Natural capital has also been evaluated by Wackernagel *et al*. on a national scale and combined with ecological footprint analyses^7^. That investigation showed that all 52 countries had more bio-capacity than they were using, thereby placing substantial pressure on natural ecosystems.

Because natural renewable resources are distributed in different geographic locations, spatial information is a crucial factor in considering both land development and resource protection. Li and Luo^8^ evaluated national sustainability combined with emergy assessment and GIS and noted that China’s sustainable development situation was not optimistic. Baroudy developed a new GIS-based model that can detect whether land is suitable for farming in Egypt^9^. Because solar is the main renewable resource in Vietnam, Polo *et al*. mapped the distribution of solar resources in Vietnam using satellite imagery^10^. Mellino *et al*. mapped the distributions of natural resource flows in the region of Campania with GRASS GIS software, offering a useful reference for governments formulating environmental policies^11^.

Previous studies have shown mapping and visualizing approach has good potentials in supporting regional environmental planning and sustainable development, however, few research has paid attention on natural renewable sources and its relationship to net primary productivity (NPP) of ecosystem with a GIS-emergy approach. Emergy, because of its original advantage, allows in comparing different types of energy and material process in one unified unit (more details about emergy is given in Methodology). Thus, a GIS-emergy approach will be quite useful in mapping, visualizing and evaluating natural renewable resources in a certain area. Japan, as an island country, has little fossil energy but abundant renewable natural resources. To know the values and distribution of renewable natural resources and NPP of ecosystem as well as their relationship will provide useful information for local environmental policy making. In this study, we chose Hokkaido as our study area, a GIS-emergy approach was employed to analysis the values for all types of renewable natural resources (including solar radiation, geothermal heat, wind, and rain) and their spatial distributions. In addition, we also estimated the values and spatial distributions of NPP of local ecosystems and analyzed the relationship between renewable natural resource flows and NPP.

## Results

**Figure 1:**
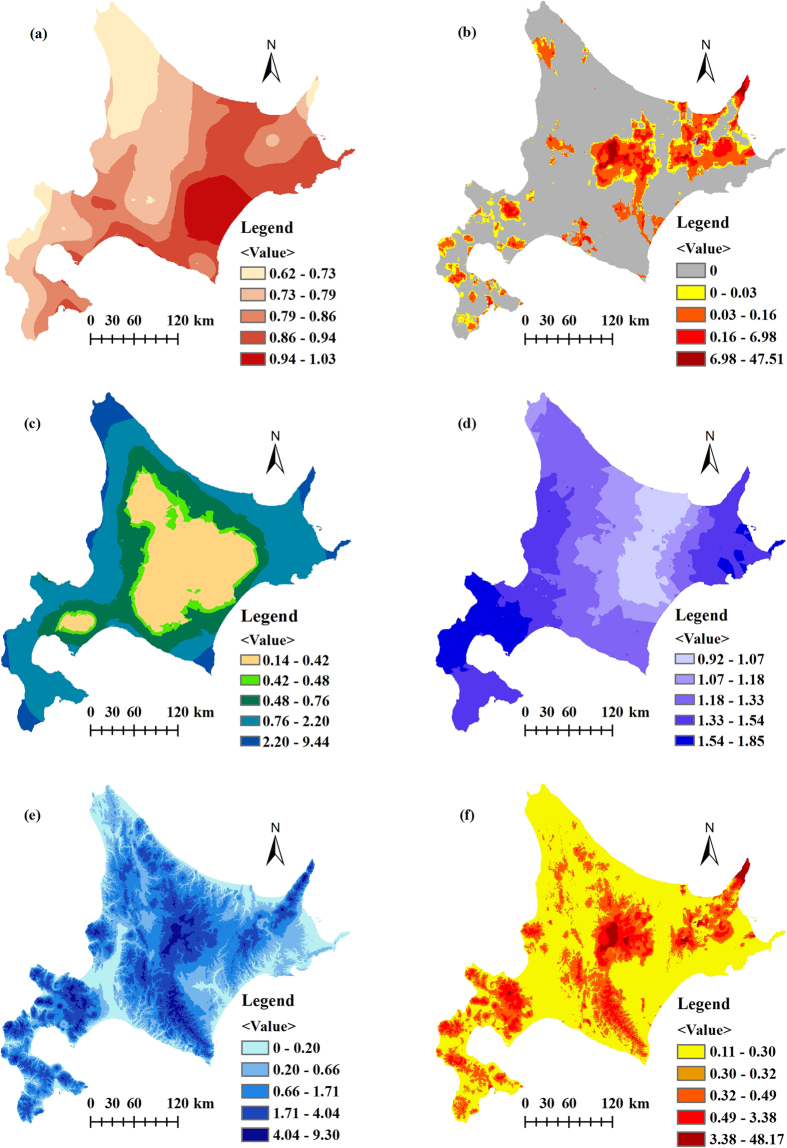
The emergy distribution of renewable natural resources in Hokkaido. (**a**) Emergy density (E+14 sej/ha/yr) of solar radiation. (**b**) Emergy density (E+16 sej/ha) of geothermal heat flow. (**c**) Emergy density (E+14 sej/ha) of wind kinetic energy. (**d**) Emergy density (E+15 sej/ha) of rainfall chemical energy. (**e**) Emergy density (E+15 sej/ha) of rainfall geopotential energy. (**f**) Emergy density (E+16 sej/ha) of renewable flows. Figure 1 was created by ArcGIS v10.2 software (Environmental Systems Research Institute, Inc, USA, version 10.2, http://www.esri.com). Data Source: Japan Meteorological Agency (http://www.jma.go.jp/jma/index.html).

The emergy distribution of renewable natural resources in Hokkaido was mapped by ArcGIS as follows (Fig. 1). Areas with certain emergy advantages can be distinguished using the visual map. In general, the emergy density of solar radiation is higher in the south than in the north Fig. 1(a) because the southern area receives sunshine for longer times than the northern area. The uneven distribution of geothermal heat flow results in uneven emergy densities of geothermal heat flows, with a very large difference in the order of magnitude Fig. 1(b). In Hokkaido, the geothermal heat flow mainly derives from volcanoes, and thus, the geothermal heat flow emergy tends to concentrate near volcanoes. The wind emergy map indicates that areas around the ocean have higher winds than areas near the center of the island Fig. 1(c). This effect is mainly due to coastal areas always having more air convection, leading to strong winds, such as typhoons. Rainfall chemical emergy maps show higher emergy values in the southwest and the northeast, both of which are near the ocean Fig. 1(d). The terrain had a major influence on rainfall geopotential energy, and thus, that map shows a different distribution trend than the rainfall chemical emergy map, although the same rainfall data were used Fig. 1(e). Figure 1(f) also shows the integrated renewable emergy flows and can be easily used to identify the total emergy values of a given area.

## Discussion

The statistical data were generated based on the emergy accounting for renewable natural resources (Table 1). The minimum value, maximum value, average value, standard deviation (SD) and total value were calculated. The SD reflects the degree to which a data set was discrete, and greater SD values usually indicate a higher degree of discreteness. Table 1 shows that geothermal heat had the largest total emergy value and a large SD. Thus, Hokkaido is rich in geothermal resources, but their distribution is uneven. According to the Ministry of Environment Government of Japan, no geothermal resources are available in most other areas, except volcanic areas^12^. Thus, the distribution of geothermal resource is linked to volcanoes. This mainly accounts for the large SD value of geothermal resources. The total emergy value of rainfall is also very large (Table 1), indicating that rainfall is relatively abundant in Hokkaido.

**Figure 2:**
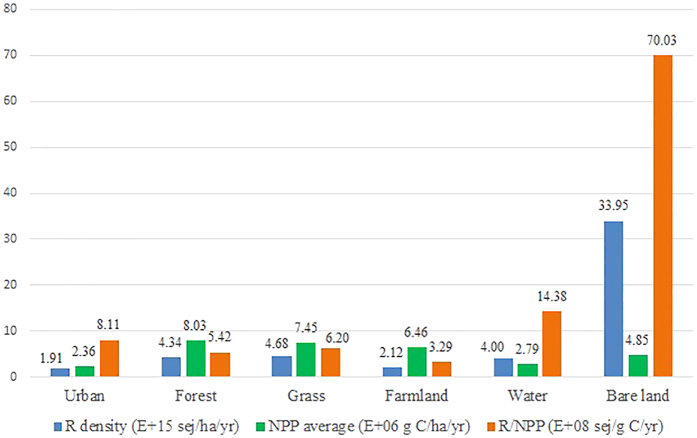
Analysis of renewable flows (R) and NPP.

We also generated a land-use map for Hokkaido (Fig. 2) to show the relationship between land-use type and renewable natural resources (Table 2). We evaluated the area of each land-use type, and found forest ecosystem and grass ecosystem occupy the largest area of Hokkaido (5.66 E+06 ha and 9.86 E+05 ha, respectively) which indicates the study area has abundant natural ecosystem. We calculated the average and gross values of renewable flows according to different land-use types. Because the average value of renewable flow was obtained from the total value divided by the area, the results reflect the density of renewable flows in a certain area. In addition, we also evaluated the NPP, which is an indicator of output of vegetation ecosystems, and associated it with land-use type (Table 2). As expected, forest area and grass area in the study area provide the largest contribution to NPP, whereas urban area contributes the minimum NPP mainly due to the vegetation ecosystem has been thoroughly disrupted in urban areas by human activities. This finding confirms the damage that urbanization causes to natural ecosystems. Because the natural ecosystem plays a vital role in sustaining human society through multiple of ecosystem services such as carbon sequestration and oxygen release, local governments should be more cautious in their policy-making.

We conducted additionally analyses to further investigate the relationship between renewable flows and NPP (Fig. 2). The highest average value of NPP was associated with vegetation ecosystems (forest and grass) which indicates the importance of vegetation in the use of renewable natural resources to produce renewable products through photosynthesis. The largest R density was not found for a vegetation ecosystem but for bare land. One important reason for the high R density in these areas is that most bare land is distributed in volcanic area. Indeed, the large geothermal value divided over a small area leads to a high R density. Those areas are not suitable for vegetation growth, however, geothermal exploitation should be encouraged by the local government.

The red bar chart labeled R/NPP in Fig. 2 was calculated by dividing the total renewable emergy by total NPP, i.e. input/output, according to land-use type. Because the results are presented in units of sej/g C, higher values of R/NPP correspond to higher required energy consumption per gram of carbon produced. This value could be used as an indicator to evaluate the productivity of different land-use types. Figure 2 shows that bare land has the largest indicator value, which corresponds to the lowest productivity. Because the main source of renewable emergy is geothermal resources on bare land, we suggest that the government should consider policies to use these resources. In areas with more vegetative cover, such as forest areas, grass land and farmland, the values of R/NPP are lower, which means a higher productivity in these areas. Renewable natural resources can be used more efficiently in these areas by the vegetation ecosystems to produce biological products. In addition, as mentioned in Millennium Ecosystem Assessment (MEA)^13^, the ecosystem services that sustain human society do not consist only of directly functional biological products, such as food or wood. Indeed, indirect functions, such as soil and water conservation, carbon sequestration, and oxygen release, are also critical to human society. In fact, natural ecosystem contribute substantially to human society. Environmental policy-making should consider protecting the vegetation ecosystems in these areas to avoid damage caused by human activities.

## Conclusion

Social-economic development benefits humans, but increasing energy demands result in resource scarcity and environmental deterioration. Renewable natural resources are considered to be a new alternative energy source and are attracting increasing attention. In this paper, we calculated the emergy values of renewable natural resources in Hokkaido and visualized their distributions. The resource distribution maps were generated at a resolution of 100 m, and areas with certain advantageous resources can easily be identified. Thus, policy recommendations regarding resource usage can be concentrated accordingly. For instance, in areas with high emergy value winds, wind-power stations should be developed, whereas solar power stations could be considered for areas rich in solar radiation emergy. Because vegetation ecosystems, such as forest and grass, store renewable natural resources, we also evaluated NPP as a measure of ecosystem renewable products. The land-use map demonstrates that natural areas have higher emergy densities and NPP than other areas, indicating that the ecosystem in these areas have a higher productivity and may also provide better ecosystem services. More accurate emergy values for renewable natural resources and their spatial distributions would constitute a useful reference for policy makers in making environmental decisions. In the case of economic exploitation which may bring damage to natural ecosystem, the R/NPP results provided in this paper can be used as an indicator and serve as a reference for a better planning and governance to minimize the impacts.

## Methodology and Materials

### Study area

Hokkaido is the second largest island in Japan and has an area of about 83457 km^2^. Unlike other places in Japan, this area experiences four distinct seasons. As the northernmost prefecture, the annual average temperature is 6–10 °C and the average annual rainfall is approximately 700–1700 mm. Hokkaido is rich in natural resources. According to the National Survey on the Natural Environment conducted by Biodiversity of Japan, the area of natural forest in Hokkaido accounts for 59.5% of the total area in Japan^14^. The main island of Hokkaido was chosen as the study area. This island has an area of 7.85E+04 km^2^, and climate data from 2015 were obtained from the Japan Meteorological Agency^15^. Data from 165 meteorological observation sites were used in this study.

The digital elevation model (DEM) data applied in this study were obtained from the Geospatial Information Authority of Japan (GSI) website^16^. As the base database, we used the 10-m mesh national base map data (JPGIS2.1, GML format). The GML files were transferred to tiff format using the transfer tool and a resampling resolution of 100 m by ArcGIS software (see Supplementary Information: Fig. 3 Digital elevation model of Hokkaido, Japan).

The land-use map of Hokkaido is presented at 100-m resolution. The original data were provided by the Japan Aerospace Exploration Agency (JAXA) and consisted of Advanced Land Observing Satellite (ALOS) high-level data^17^. The final data were updated in February 2016. Throughout this study, land-use is reclassified as urban (refers to constructed area), forest, grass, farmland, bare land and water (see Supplementary Information: Fig. 4 Land-use and land-cover map of Hokkaido, Japan).

### Emergy-GIS approach

Emergy analysis was originally proposed by Odum on the theoretical basis that all types of materials and energy are mainly derived from solar energy^18^. To simplify the fact that different types of materials and energy possess different properties and are expressed in different units, emergy theory introduces the concept of transformity. Transformity allows all of the various properties to be measured in terms of the same unit, called solar equivalent joules (sej). Emergy analysis is a powerful environmental accounting method that has been used in many ecological studies^19^,^20^,^21^,^22^,^23^.

GIS is a powerful spatial analysis tool that is widely used today. The development of remote-sensing technology has made it possible to study data on a large scale over long term, satisfying the demand for spatial-temporal information in scientific studies^24^,^25^,^26^.

Because emergy analysis is a relatively accurate environmental accounting method and because GIS can provide visualizable spatial information, an emergy-GIS framework is employed in this study.

### Emergy map of solar radiation

We generated a distribution solar radiation map using a kriging interpolation tool with a resolution of 100 m and meteorological statistical data in ArcGIS. The formula is as follows:


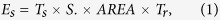


where *E*_*s*_ is the emergy of solar radiation, *T*_*s*_ is the sunshine time, *S.* is the solar irradiance (here, 1360.8 W/m^2^ according to Kopp’s study)^27^, and *T*_*r*_ is the emergy transformity of solar radiation, which is equal to 1 sej/j^18^.

### Emergy map of geothermal heat flow

Geothermal energy is a clean and sustainable resource generated by the Earth. Some countries use it for cooking or heating. As science and technology have developed, geothermal resources are used in different ways, such as to generate electricity. Because the Hokkaido region is located on a mountainous island in the border area of the Eurasian plate and the Pacific plate, which is also called the ring of fire, volcano and earthquake activity is frequent. The original geothermal data for this study area were obtained from the Ministry of Environment, Government of Japan^12^, and contained three map layers divided by temperature: 53 °C–120 °C, 120 °C–150 °C, and more than 150 °C. The unit of value is expressed in kw/km^2^. We used the overlay analysis tool in ArcGIS to generate a composite layer. The emergy value of the geothermal heat flow was computed by using Eq. (2):


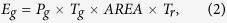


where *P*_*g*_ is the geothermal power; *T*_*g*_ is the time period (one year, equal to 3.15E+07s), and *T*_*r*_ is the emergy transformity of solar radiation (2.03E+04 sej/j)^28^.

### Emergy map of wind kinetic energy

The unit of the wind meteorological statistical data is m/s. We obtained wind speed for all of Hokkaido by means of a kriging interpolation tool. The emergy value of the wind kinetic energy is calculated using Eq. (3):


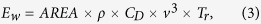


where *ρ* is the air density (1.3 kg/m^3^), *C*_*D*_ is the drag coefficient (1.00E−03), *v* is the wind speed, and *T*_*r*_ is the emergy transformity of the wind kinetic energy (2.45E+03 sej/j)^29^.

### Emergy map of rainfall chemical and emergy map of rainfall geopotential

The two maps of rainfall chemical and rainfall geopotential were both derived from precipitation data. First, the precipitation map was generated using the kriging interpolation tool in ArcGIS. Then, the emergy map of rainfall chemical was obtained using Eq. (4):


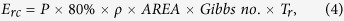


where P is the yearly precipitation measured in mm, the evapotranspiration rate is 20%, *ρ* is the density of water (1.00E+03 kg/m^3^), the Gibbs no. is the Gibbs free energy (4.94 j/g), and *T*_*r*_ is the emergy transformity of rainfall chemical (3.05E+04 sej/j)^29^.

Because the rainfall geopotential is closely related to elevation, the DEM model is needed to evaluate the rainfall geopotential energy. The formula is as follows:


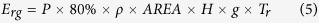


where P is the yearly precipitation measured in mm, the evapotranspiration rate is 20%, ρ is the density of water (1.00E+03 kg/m^3^), H is the elevation obtained from the DEM model, g is the acceleration of gravity, and *T*_*r*_ is the emergy transformity of rainfall geopotential (4.70E+04 sej/j)^29^.

### Emergy map of renewable flows

Because the solar radiation, geothermal, wind kinetic and precipitation are all evaluated in emergy and presented as maps, we integrated the renewable natural resources of Hokkaido into a synthesis map using the overlay tool in ArcGIS.

### NPP and ecosystem services

NPP is defined as the amount of net useful chemical materials produced by vegetation in an ecosystem (i.e., primary producers) and is usually expressed as g carbon/m^2^/yr. Human life cannot be sustained without plant photosynthesis absorbing carbon dioxide, releasing oxygen, and generating organic matter. The primary producer is so important for human society that it is the root of all four types of ecosystem services: provisioning, regulating, supporting, and cultural. Provisioning services provide food and the organic material produced by plants for human society. Regulating services promote carbon sequestration and oxygen release. Supporting services support soil and water conservation and biodiversity protection. Cultural services offer recreational activities^13^. NPP is so closely connected with primary producers that it could be considered as the foundation of ecosystem services. Thus, NPP has been widely used as a key indicator to reflect the ecosystem productivity^13^,^30^,^31^,^32^,^33^.

The NPP data applied in this study were derived from MOD17A3 Version 6 product^34^ in units of kg carbon/m^2^/yr. In this product, preprocessing, including projection transformation and extraction, is necessary. Data values are obtained after these processing (see Supplementary Information: Fig. 5 NPP of Hokkaido, Japan).

## Additional Information

**How to cite this article**: Wang, C. *et al*. Evaluating renewable natural resources flow and net primary productivity with a GIS-Emergy approach: A case study of Hokkaido, Japan. *Sci. Rep.* **6**, 37552; doi: 10.1038/srep37552 (2016).

**Publisher’s note:** Springer Nature remains neutral with regard to jurisdictional claims in published maps and institutional affiliations.

## Supplementary Material

Supplementary Information

## Figures and Tables

**Table 1:** Statistical emergy values of renewable natural resources.

Emergy maps (E+14 sej/ha/yr)	Min.	Max.	Average	SD	Total (E+20 sej/yr)
Solar radiation	0.62	1.03	0.81	0.08	6.44
Geothermal heat flow	0.00	4751.62	16.33	136.84	128.48
Wind kinetic energy	0.14	9.44	0.91	0.77	7.21
Rainfall, chemical potential energy	9.27	18.56	12.93	1.74	101.77
Rainfall, geopotential energy	0.00	93.01	11.97	11.24	94.17
Renewable flows	11.55	4817.31	42.98	139.73	337.97

**Table 2:** The distribution of renewable flows (R) and NPP through different land-use type.

Land-use type	Area (ha)	R-average (E+15 sej/ha/yr)	R (E+20 sej/yr)	R land-use type/R total (%)	NPP-average (E+06 g C/ha/yr)	NPP (E+11 g C/yr)	NPP land-use type/NPP total (%)	R/NPP (E+08 sej/g C/yr)
Urban	1.42E+05	1.91	2.72	0.80	2.36	3.35	0.57	8.11
Forest	5.66E+06	4.34	246.45	72.92	8.03	454.62	76.18	5.42
Grass	9.86E+05	4.68	46.23	13.67	7.45	73.44	12.32	6.20
Farm	9.02E+05	2.12	19.20	5.68	6.46	58.29	9.76	3.29
Water	1.07E+05	4.00	4.30	1.27	2.79	2.99	0.51	14.38
Bare land	5.59E+04	33.95	18.98	5.61	4.85	2.71	0.46	70.03
Total (Hokkaido Region)	7.85E+06	4.29	337.97		7.59	595.40		5.67
