# Supplementary material for: Evaluating renewable natural resources flow and net primary productivity with a GIS-Emergy approach: A case study of Hokkaido, Japan
Source: Sci Rep. 2016 Nov 18;6:37552. doi: 10.1038/srep37552 (PMC5114597; doi:10.1038/srep37552)
Supplement: Supplementary Information [file srep37552-s1.doc]

**Evaluating renewable natural resources flow and net primary productivity with a GIS-Emergy approach: A case study of Hokkaido, Japan**

Chengdong Wang^a,b^, Shenyan Zhang^b^, Wanglin Yan^b^, Renqing Wang^a^, Jian Liu^c^, Yutao Wang^a,d*^

**Fig. 3 Digital elevation model of Hokkaido, Japan**

**
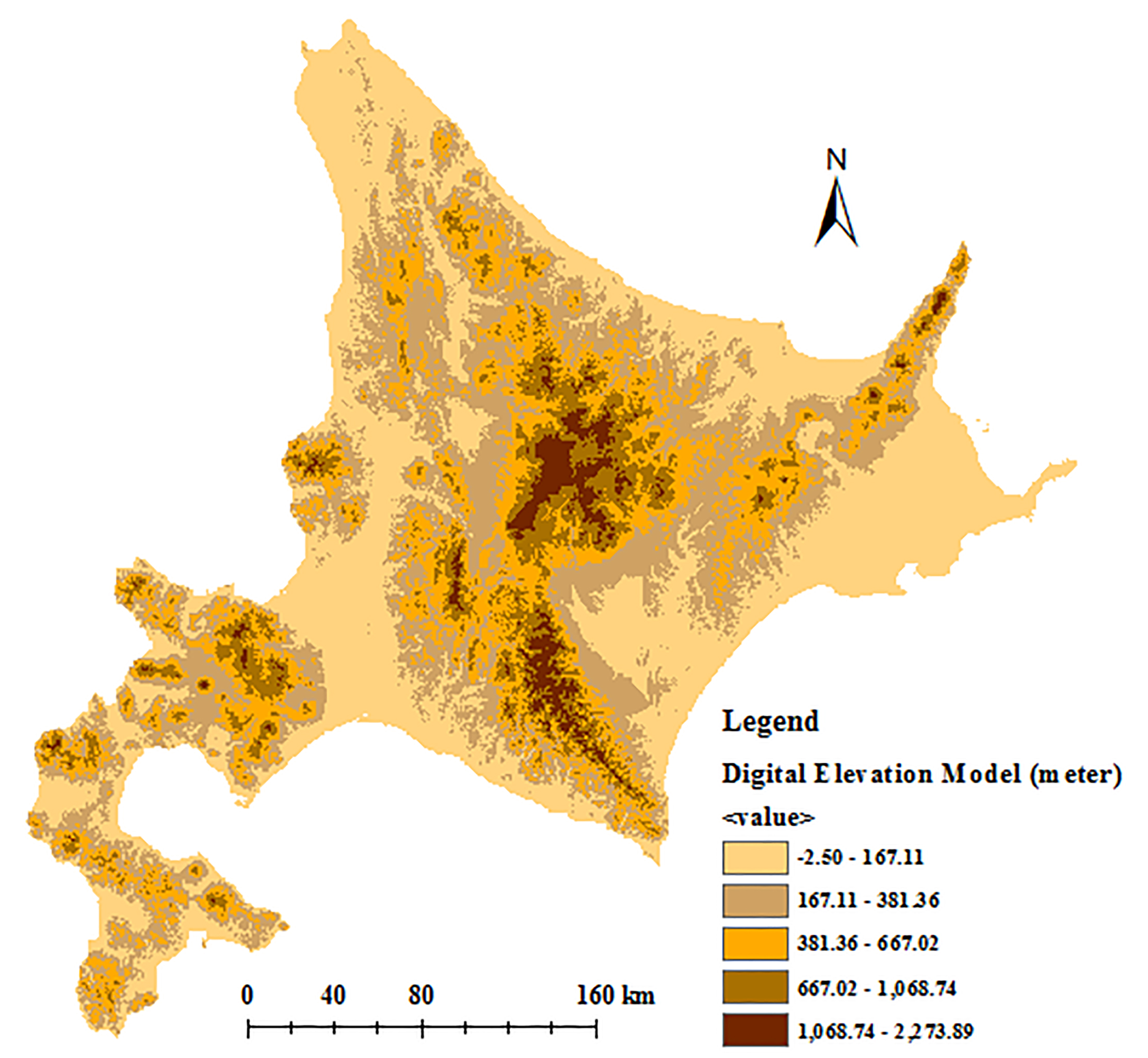
**

**Fig. 3** was created by ArcGIS v10.2 software (Environmental Systems Research Institute, Inc, USA, version 10.2, http://www.esri.com). Data Source: Geospatial Information Authority of Japan (GSI) website ([http://www.gsi.go.jp](http://www.gsi.go.jp/)).

**Fig. 4 Land-use and land-cover map** **of Hokkaido, Japan**

**
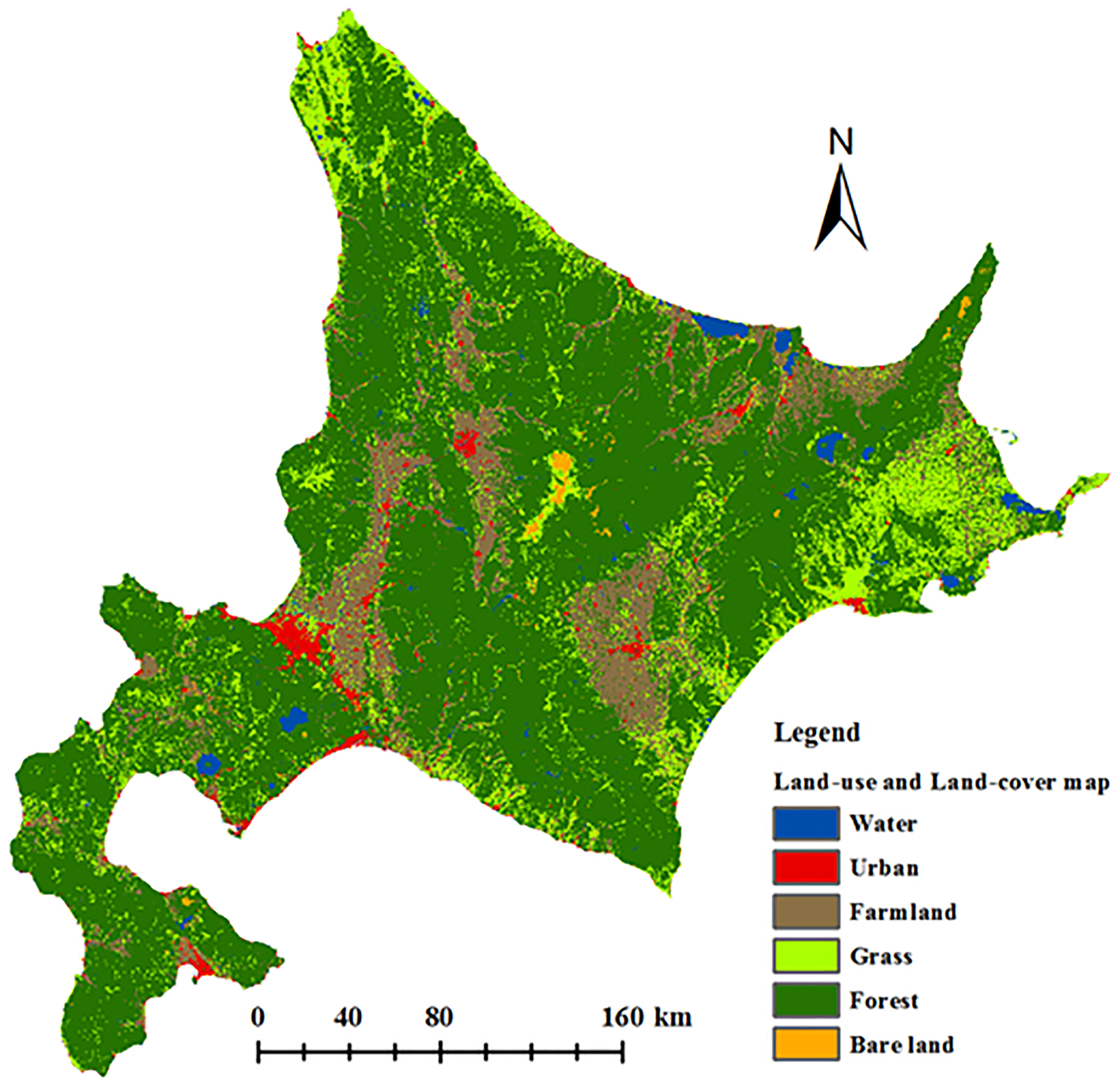
**

**Fig. 4** was created by ArcGIS v10.2 software (Environmental Systems Research Institute, Inc, USA, version 10.2, http://www.esri.com). Data Source: Japan Aerospace Exploration Agency (JAXA) website (<http://www.eorc.jaxa.jp/ALOS/lulc/lulc_jindex.htm>).

**Fig. 5 NPP of Hokkaido, Japan**

**
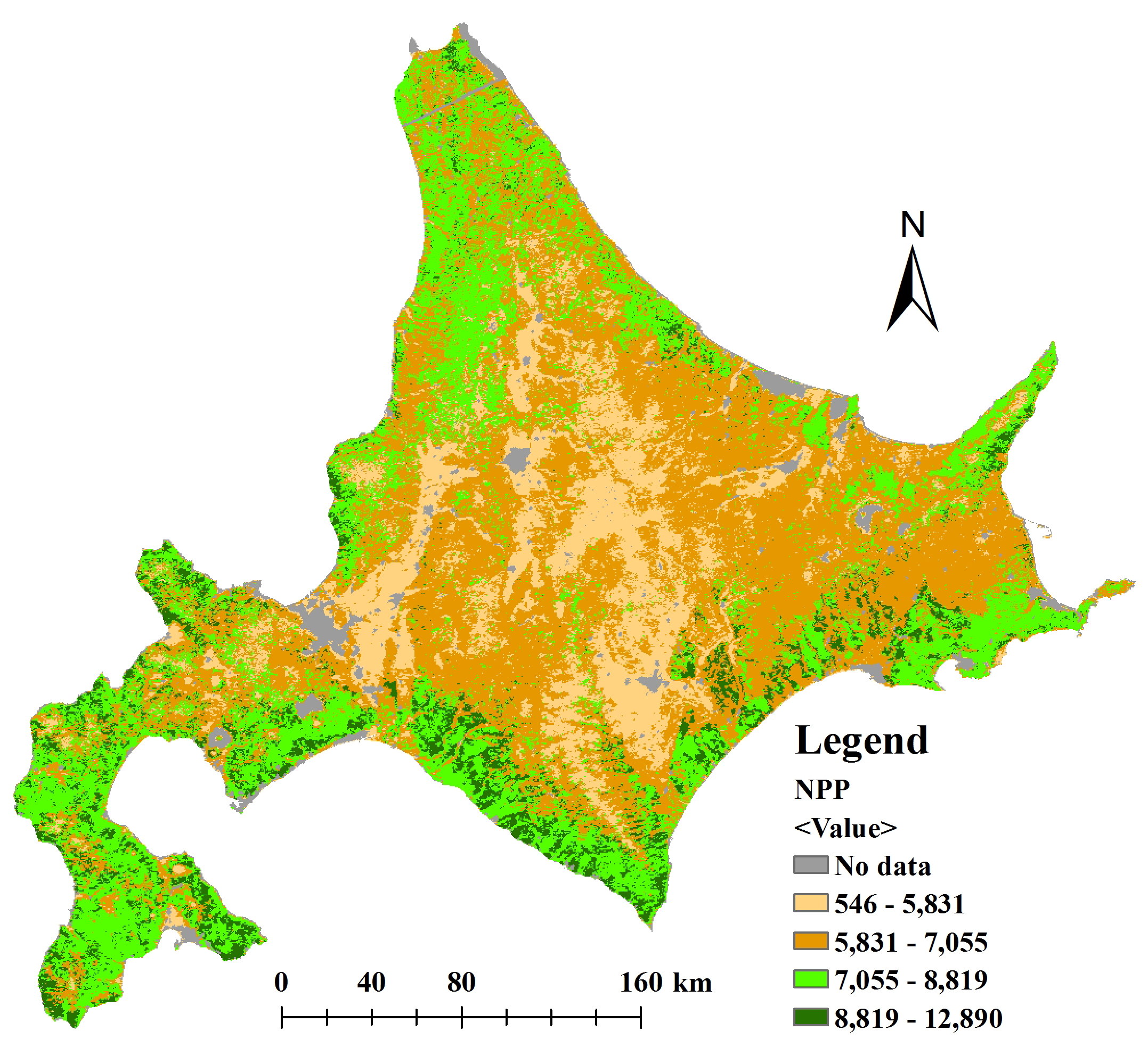
**

**Fig. 5** was created by ArcGIS v10.2 software (Environmental Systems Research Institute, Inc, USA, version 10.2, http://www.esri.com). Data Source: S. Running, Q. Mu, M. Zhao. (2015). MOD17A3H MODIS/Terra Net Primary Production Yearly L4 Global 500m SIN Grid V006. NASA EOSDIS Land Processes DAAC (<http://doi.org/10.5067/MODIS/MOD17A3H.006>).
